# Macrophage miR-149-5p induction is a key driver and therapeutic target for BRONJ

**DOI:** 10.1172/jci.insight.159865

**Published:** 2022-08-22

**Authors:** Xin Shen, Weiwen Zhu, Ping Zhang, Yu Fu, Jie Cheng, Laikui Liu, Rongyao Xu, Hongbing Jiang

**Affiliations:** 1 Jiangsu Key Laboratory of Oral Diseases and; 2 Department of Oral and Maxillofacial Surgery, Affiliated Hospital of Stomatology, Nanjing Medical University, Nanjing, China.; 3 Jiangsu Province Engineering Research Center of Stomatological Translational Medicine, Nanjing, China.; 4 Department of Basic Science of Stomatology, Affiliated Hospital of Stomatology, Nanjing Medical University, Nanjing, China.

**Keywords:** Bone Biology, Bone disease

## Abstract

Bisphosphonate-related (BP-related) osteonecrosis of the jaw (BRONJ) is one of the severe side effects of administration of BPs, such as zoledronic acid (ZA), which can disrupt the patient’s quality of life. Although the direct target of skeletal vasculature and bone resorption activity by BPs has been phenomenally observed, the underlying mechanism in BRONJ remains largely elusive. Thus, it is urgently necessary to discover effective therapeutic targets based on the multifaceted underlying mechanisms in the development of BRONJ. Here, we determined the inhibitory role of ZA-treated macrophages on osteoclast differentiation and type H vessel formation during tooth extraction socket (TES) healing. Mechanistically, ZA activated the NF-κB signaling pathway and then induced p65 nuclear translocation in macrophages to promote miR-149-5p transcription, resulting in impaired osteoclast differentiation via directly binding to the *Traf6* 3′-UTR region. Moreover, we identified that miR-149-5p–loaded extracellular vesicles derived from ZA-treated bone marrow–derived macrophages could regulate biological functions of endothelial cells via the Rap1a/Rap1b/VEGFR2 pathway. Furthermore, local administration of chemically modified antagomiR-149-5p was proven to be therapeutically effective in BRONJ mice. In conclusion, our findings illuminate the dual effects of miR-149-5p on skeletal angiogenesis and bone remolding, suggesting it as a promising preventive and therapeutic target for BRONJ.

## Graphical abstract

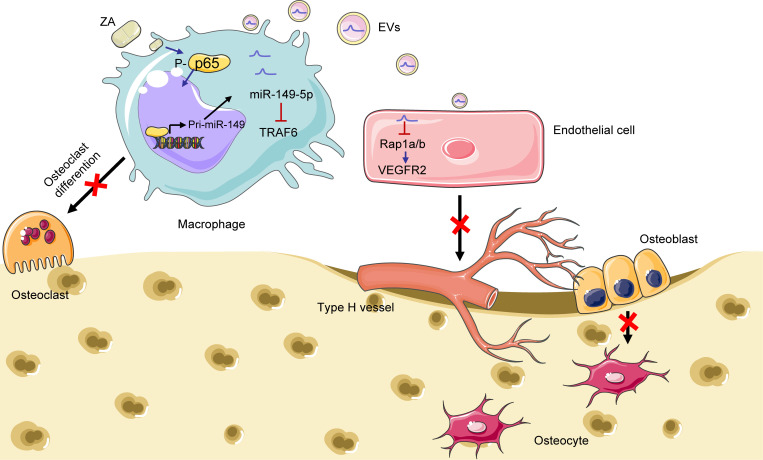

## Introduction

Bisphosphonates (BPs) are widely used to reduce risk of osteoporotic fracture; they act by reducing the rate of bone resorption and bone remodeling (1, 2). Certain BPs have been routinely prescribed to prevent skeletal complications in patients with multiple myeloma and malignant tumor bone metastasis (3, 4). However, administration of a high dose of intravenous BPs, such as zoledronic acid (ZA), may cause BP-related osteonecrosis of the jaw (BRONJ), one of the rare adverse effects characterized by necrotic bone exposure in the maxillofacial region for more than 8 weeks in patients who received or are currently receiving BPs, with no history of radiation treatment (5–7). Oral operations such as tooth extraction and inflammatory dental diseases are known as the leading predisposing factors for development of BRONJ (8, 9). Following the first report of BRONJ by Marx in 2003 (10), more and more evidence has indicated that inflammatory dental diseases lead to the death of alveolar bone tissue. Osteoclastic activity removes necrotic bone tissue, which occurs in patients not taking BPs. This activity is slowed in patients taking BPs, with necrotic bone accumulating and exposed to the oral cavity (11–14). However, BRONJ pathophysiology has many gray areas that remain unexplored. Therefore, understanding disease pathophysiology is crucial to strategizing effective therapeutic regimens to treat BRONJ and simultaneously improve the quality of life of patients.

Efficient blood supply is essential for overall skeletal integrity and during the healing process after bone trauma; hence, aberrant changes in the bone microvasculature may be detrimental, leading to osteonecrosis (15, 16). Previous studies have found that BPs, especially nitrogen-containing BPs, can drastically decrease the blood vessel count in the affected tissues (17). Furthermore, it has been shown that BPs can inhibit endothelial cell (EC) migration and proliferation by inducing oxidative stress, leading to impaired bone repair (18). Type H vessels, defined by dual expression of CD31 and endomucin (CD31^hi^EMCN^hi^), have been found to participate in the coupling of osteogenesis and angiogenesis during skeletal development (19–21). Notably, a recent study has reported that type H vessels also exist in adult alveolar bone and play an important role in alveolar bone remodeling (22). Hence, we raised a hypothesis indicating that administration of ZA could disrupt skeletal angiogenesis, impeding the healing of jaw bone in BRONJ.

Macrophages, found in almost all tissues, are crucial for osteoimmunology and skeletal homeostasis (23–25). In a previous study, we verified that ZA could promote TLR-4–mediated proinflammatory M1 macrophage polarization in BRONJ (26). Moreover, the close association between the spatial distribution of activated macrophages and newly forming vessels has been observed during the bone callus formation and periosteal regeneration (27, 28). Extracellular vesicles (EVs) are small secretory vesicles involved in intercellular communication and cellular differentiation. EVs reportedly carry diverse genetic elements such as miRNAs (29–31). miRNAs, acting as small noncoding RNAs (22–25 nucleotides) and key posttranscriptional modulators, play pivotal roles in cell-to-cell communication and bone regeneration (32, 33). Given that miRNAs are indispensable in cross-cellular gene regulation, we aimed to investigate whether ZA could alter the communication signaling between the activated macrophages and ECs via shedding miRNA-loaded EVs in BRONJ.

Here, we showed that ZA treatment could disrupt the abundance of type H vessels in a tooth extraction socket (TES) in a mouse model of BRONJ for the first time to our knowledge. We not only identified miR-149-5p, which is abundantly found in ZA-treated macrophage-derived EVs, as the key modulator of EC biological functions via the Rap1a/Rap1b/VEGFR2 pathway, but we also verified miR-149-5p binding to the 3′-UTR of TNF receptor–associated factor 6 (*Traf6*) gene and found that miR-149-5p impeding osteoclastic differentiation. To further explore the therapeutic potential of miR-149-5p, we used a local injection system to confirm that inhibition of miR-149-5p could promote skeletal angiogenesis and bone remolding in TES. Taken together, these findings indicate that miR-149-5p may act as a potential preventive and therapeutic target for BRONJ.

## Results

### The BRONJ mouse model shows decreased osteoclast activity and impaired skeletal angiogenesis.

**Figure 1:**
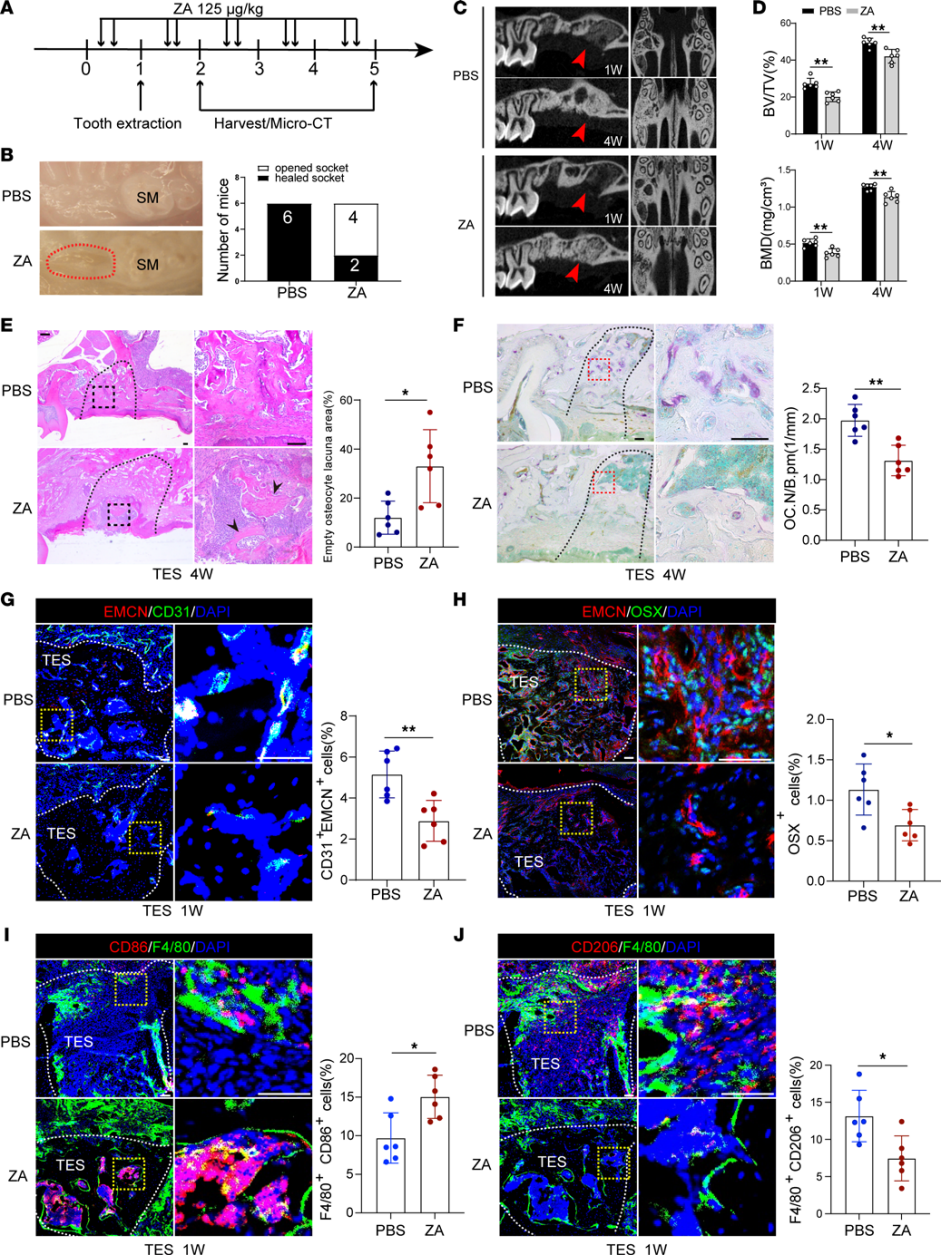
BRONJ mouse model presents decreased osteoclast activity and impaired skeletal angiogenesis. (**A**) Schedule of the establishment of the BRONJ mouse model. Numbers indicate treatment days. (**B**) Clinical appearance of the gingival mucosa at TES. Red dotted lines outline the open socket adjacent to the second molar (SM). The number of mice with the open socket was quantified. (**C**) Micro-CT analysis of new bone fill in TES. (**D**) Quantitative measurements of BV/TV and BMD for new bone in TES. (**E**) Representative H&E-stained images of TES 4 weeks after tooth extraction. Scale bars: 100 μm. *n* = 6. (**F**) Representative TRAP-stained images showing osteoclastic activity and quantification of osteoclast numbers in TES. Scale bars: 100 μm. *n* = 6. (**G**) Representative immunostained images of EMCN^hi^CD31^hi^ vessels in TES from control and BRONJ mice. Scale bars: 100 μm. *n* = 6. (**H**) Representative immunostained images of osterix^+^ perivascular osteoprogenitors. Scale bars: 100 μm. *n* = 6. (**I** and **J**) Representative immunostained images of F4/80^+^CD86^+^ (M1) or F4/80^+^CD206^+^ (M2) macrophages. Scale bars: 100 μm. *n* = 6. Results are presented as the mean ± SD. *P < 0.05; **P < 0.01 by Student’s *t* test.

To investigate the potential pathomechanism of BRONJ, we established a BRONJ-like mouse model by extracting the first molar teeth in mice to mimic BRONJ lesions in humans (Figure 1A). Maxillae of both mice treated with PBS- (control) and ZA-treated mice were collected, and TES healing was analyzed. PBS-treated mice showed complete oral socket wound healing (6 of 6 mice), whereas 66.7% of ZA-treated mice (4 of 6 mice) exhibited open extraction sockets (Figure 1B). Our microcomputed tomography (micro-CT) analysis indicated significant reductions in both bone volume fraction (BV/TV) and bone mineral density (BMD) in the TES region of ZA-treated mice, compared with those of control mice (Figure 1, C and D). Furthermore, more obvious socket necrotic bone area covered by incomplete oral mucosa could be observed in ZA-treated mice (Figure 1E). Tartrate-resistant acid phosphatase (TRAP) staining showed a marked decline in the number of osteoclasts in the TES region in ZA-treated mice compared with that in their control counterparts (Figure 1F).

It has been reported that BPs are associated with a decline in bone microvasculature (17, 18). However, little is known about the underlying mechanism of how BPs, such as ZA, disrupt skeletal angiogenesis maintenance in jaw bone. Immunolabeling of TES sagittal cross-sections revealed a significant reduction in the abundance of type H vessels in ZA-treated mice compared with PBS-treated mice; this was quantified by the density of CD31^hi^EMCN^hi^ vessels (Figure 1G). We next measured osterix (OSX) expression to analyze the comparative abundance of osteoprogenitors in ZA-treated and control mice; there was a significant reduction in OSX level in ZA-treated mice (Figure 1H). We also evaluated macrophage polarization and found that ZA could promote M1 polarization (F4/80^+^CD86^+^) and inhibit M2 polarization (F4/80^+^CD206^+^) of macrophages in TES (Figure 1, I and J), results consistent with those in one of our previous studies (26). Taken together, we concluded that ZA administration might lead to attenuated osteoclast activity and impaired skeletal angiogenesis in BRONJ.

### ZA-treated bone marrow–derived macrophages impede biological functions of ECs via secreting EVs.

**Figure 2:**
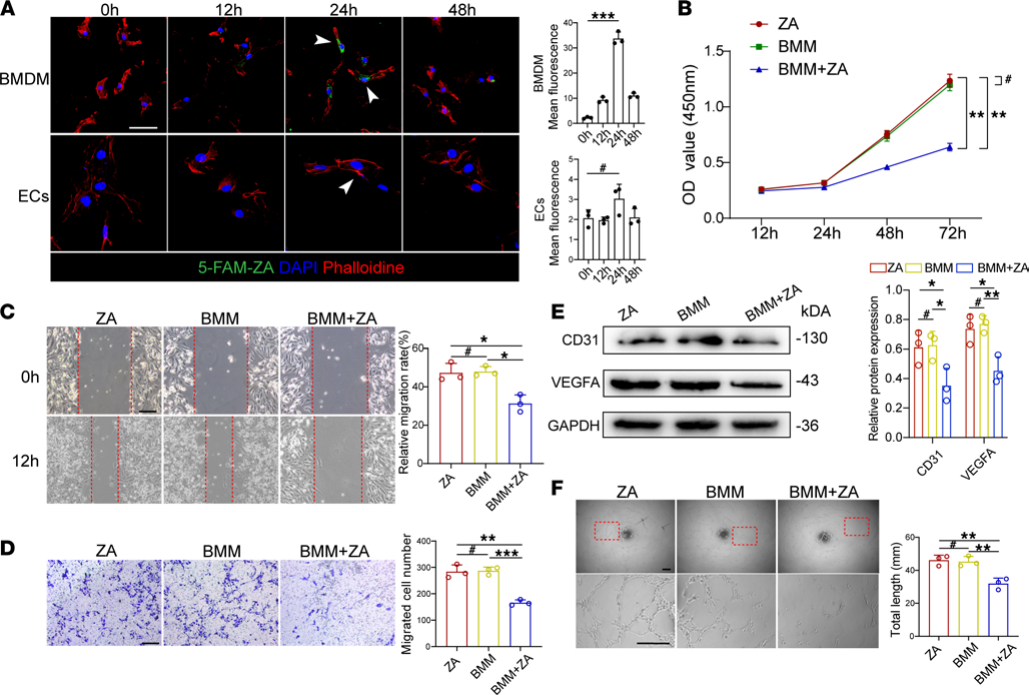
ZA-treated BMMs impede biological functions of ECs. (**A**) Representative immunostained images showing bone marrow–derived macrophages (BMMs) and ECs treated with 5-FAM-ZA (10 μM) for the indicated time and quantification analysis. Scale bars: 20 μm. *n* = 3. (**B**) CCK8 assay analysis showing EC proliferation after direct treatment with ZA, coculture with BMMs, or coculture with BMMs treated with ZA. *n* = 3. (**C**) Representative images of wound scratch assay of ECs and quantification analysis. Scale bars: 100 μm. *n* = 3. (**D**) Representative images of Transwell migration assay of ECs and quantification analysis. Scale bars: 100 μm. *n =* 3. (**E**) Western blot and semiquantitative analysis of EC CD31 and VEGF expression. *n* = 3. (**F**) Representative images of tube formation assay of ECs and quantification analysis. Scale bars: 100 μm. *n* = 3. Results are presented as the mean ± SD. *P < 0.05; **P < 0.01; ***P < 0.001; ^#^*P* > 0.05 by 1-way ANOVA.

To investigate whether BPs could directly affect ECs or bone marrow–derived macrophages (BMMs), cells were treated with fluorophore-tagged ZA analog 5-FAM-ZA (10 μM), followed by immunofluorescence analysis. We found significantly higher green signals in BMMs than in ECs, indicating BMM’s higher uptake capacity for ZA (Figure 2A). Considering the key roles of macrophages in the bone regeneration process, ECs were treated with supernatant from BMMs or ZA-treated BMMs to examine the effect of BMMs on biological functioning of ECs. The CCK8 assay exhibited declined cell proliferation ability of ECs when cocultured with supernatant from BMMs treated with 10 μM ZA (Figure 2B). Moreover, we found that ZA-treated BMMs not only attenuated the migration ability of ECs (Figure 2, C and D) but also inhibited EC expression of CD31 and VEGFA, angiogenesis-related proteins (Figure 2E). Finally, the tube formation assay revealed that ZA-treated BMMs significantly inhibited the tube formation ability of ECs (Figure 2F). These results reveal the inhibitory role of ZA-treated BMMs on ECs.

**Figure 3:**
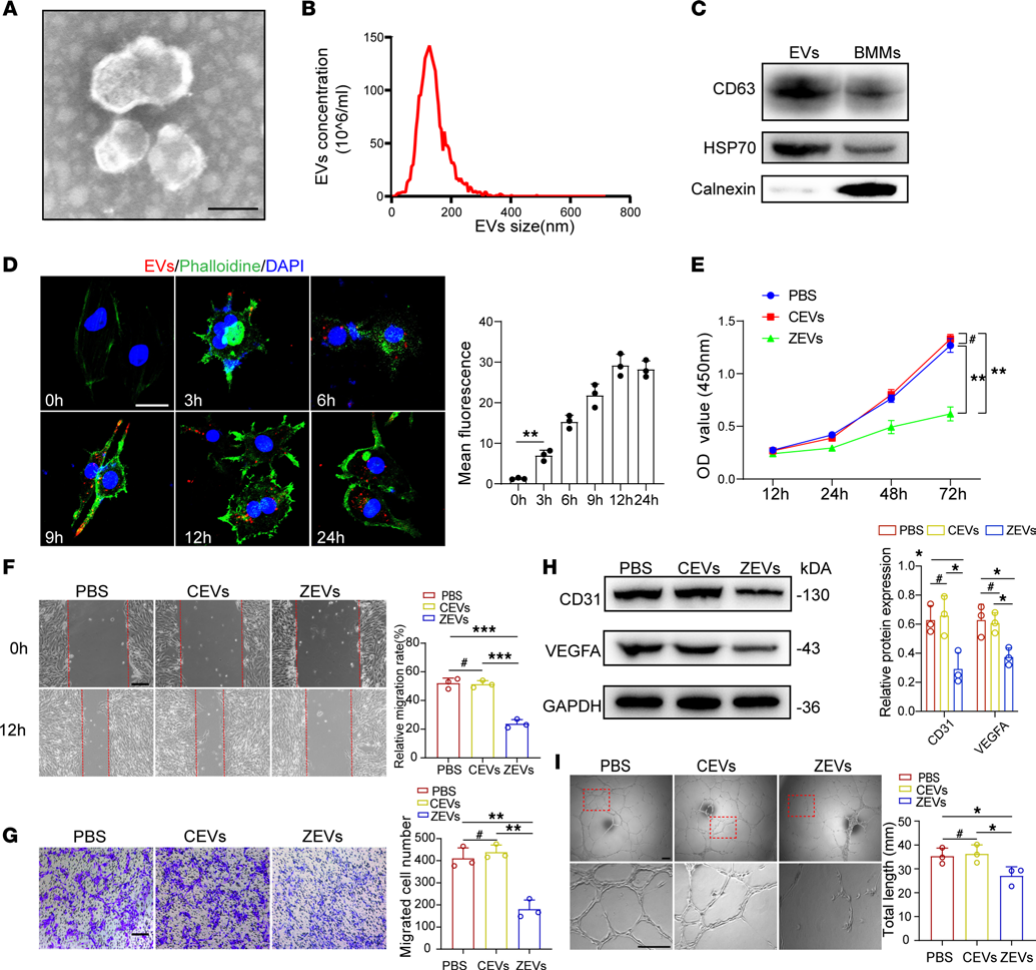
Extracellular vesicles derived from ZA-treated BMMs inhibit biological functions of ECs. (**A**) A representative image of extracellular vesicles (EVs) from bone marrow–derived macrophages (BMMs) by transmission electron microscopy. Scale bar: 100μm. (**B**) Nanoparticle tracking analysis of EVs for calculating the size distribution. The *y* axis indicates particle number concentration, and the *x* axis indicates particle size. (**C**) CD63, HSP70, and calnexin from EVs and BMMs were detected by Western blot. (**D**) Representative immunostained images showing DiI-labeled EVs from BMMs entering ECs that were cocultured for the indicated time. Scale bars: 20 μm. *n* = 3. (**E**) CCK8 assay analysis showing EC proliferation after coculture with BMM-derived EVs. *n* = 3. (**F**) Representative images of wound scratch assay of ECs and quantification analysis. Scale bars: 100 μm. *n* = 3. (**G**) Representative images of Transwell migration assay of ECs and quantification analysis. Scale bars: 100 μm. *n =* 3. (**H**) Western blot and semiquantitative analysis of CD31 and VEGF expression of ECs. *n* = 3. (**I**) Representative images of tube formation assay of ECs and quantification analysis. CEVs, control BMM-derived EVs; ZEVs, ZA-treated, BMM-derived EVs. Scale bars: 100 μm. *n* = 3. Results are presented as the mean ± SD *P < 0.05; **P < 0.01; ***P < 0.001; ^#^*P* > 0.05 by 1-way ANOVA.

Considering EVs as important carriers of intercellular communication, we next set out to explore the potential communication mechanism between BMMs and ECs. EVs from PBS- or ZA-treated BMMs were purified and analyzed under transmission electron microscopy (Figure 3A). The size distribution of EVs was examined by nanoparticle tracking analysis (Figure 3B). EV markers CD63 and HSP70 were found to be highly expressed in BMM-derived EVs when calnexin (a cytosolic marker protein) was almost undetectable (Figure 3C). We next confirmed that EVs could shuttle from BMMs to ECs using lipophilic tracer dialkylcarbocyanine-labeling (DiI-labeling) of EVs by coculturing of BMMs and ECs (Figure 3D). CCK8 assay results showed that EVs derived from ZA-treated BMMs significantly inhibited the proliferation ability of ECs (Figure 3E). Wound scratch and Transwell migration assays further confirmed the effect of EVs derived from ZA-treated BMMs on preventing EC migration (Figure 3, F and G). Furthermore, Western blot and tube formation assay revealed that EVs derived from ZA-treated BMMs inhibited CD31 and VEGFA expression and the tube formation ability of ECs (Figure 3, H and I). Overall, these data support the notion that EVs derived ZA-treated BMMs impair the biological functions of ECs.

### ZA activates p65 nuclear translocation to promote miR-149-5p transcription and loading in BMM-derived EVs.

**Figure 4:**
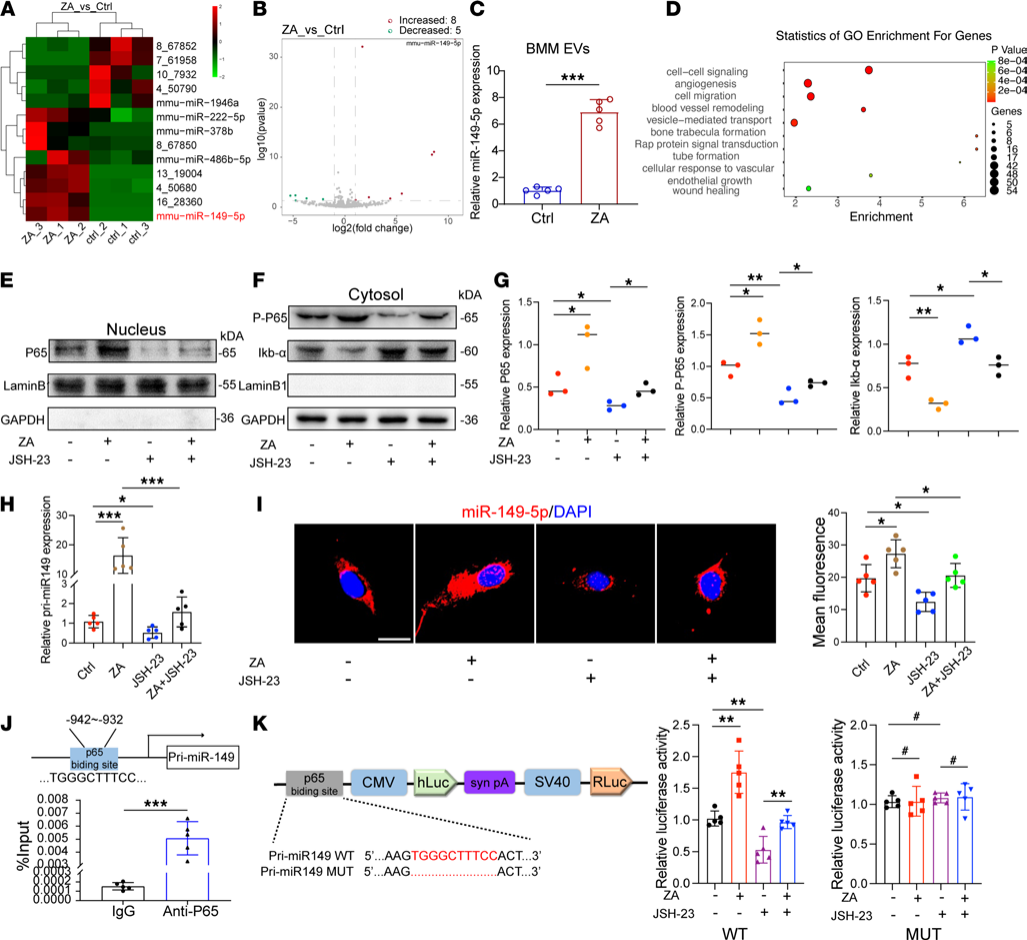
ZA activates p65 nuclear translocation to promote miR-149-5p transcription and loading in BMM-derived EVs. (**A**) Unsupervised hierarchical clustering and heatmap of the differential expression of miRNAs in bone marrow–derived macrophage–derived (BMM-derived) EVs. (**B**) Volcano plot showing differential miRNAs. (**C**) qRT-PCR analysis of relative expression of miR-149-5p from BMM-derived EVs. *n* = 5. (**D**) Gene ontology (GO) enrichment bubble plot of target genes of miR-149-5p in biological processes. (**E**) Western blot analysis of p65 from the nuclear extracts after ZA or JSH-23 treatment. (**F**) Western blot analysis of p65 phosphorylation (P-P65) and ikb-α in the cytoplasmic extracts after ZA or JSH-23 treatment. (**G**) Semiquantitative analysis of Western blot in **E** and **F**. *n* = 3. (**H**) qRT-PCR analysis of relative expression of pri-miR149 in BMMs. *n* = 6. (**I**) Distribution of miR-149-5p in BMMs as labeled by FISH assay. Scale bars: 20 μm. (**J**) ChIP analysis of p65 binding to the putative region of pri-miR149 promoter. *n* = 5. (**K**) WT and mutation vectors were constructed for dual-luciferase reporter assays to confirm the result that p65 binds to the putative region of pri-miR149 promoter. *n* = 5. Results are presented as the mean ± SD. *P < 0.05; **P < 0.01; ***P < 0.001; ^#^*P* > 0.05 by Student’s *t* test or 1-way ANOVA.

Due to the diverse compositions of miRNAs in EVs, based on their origin and biological functions, we performed miRNA-sequencing (miRNA-Seq) analysis of total RNA isolated from BMM-derived EVs to identify candidate miRNAs that potentially participate in the communication between BMMs and ECs. Among the identified miRNAs, 5 miRNAs were significantly downregulated, while 8 miRNAs were significantly upregulated in 10 μM ZA-treated BMM-derived EVs compared with controls. Based on these results, we focused on miR-149-5p, which was most obviously upregulated in the ZA-treated group (Figure 4, A and B). It has been reported that miR-149-5p is significantly overexpressed (8-fold) in peripheral lymphoid of patients with BRONJ (34) and a determinant of bone mass (35). We further verified an increased miR-149-5p in EVs derived from ZA-treated BMM by qRT-PCR (Figure 4C). Gene ontology enrichment analysis indicated that miR-149-5p target genes were mainly associated with critical biological processes, including cell-cell signaling, angiogenesis, and blood vessel remodeling (Figure 4D). Moreover, 6 previously reported dysregulated miRNAs (34) were also detected along with miR-149-5p in our BRONJ mouse sera by qRT-PCR analysis (Supplemental Figure 1; supplemental material available online with this article; https://doi.org/10.1172/jci.insight.159865DS1).

One of our previous studies showed that ZA activates the TLR-4 pathway and its downstream NF-κB signaling in macrophages (26). In this study, we further showed that ZA (10 μM) increased the p65 level in the nucleus and p65 phosphorylation in the cytoplasm and that this could be reversed by JSH-23 (10 μM), an aromatic diamine compound with the special inhibitory effect on nuclear translocation of p65 (Figure 4, E–G). To investigate whether the transcription factor p65 could transcriptionally regulate miR-149-5p expression, we used a FISH assay to detect the cellular location and expression of miR-149-5p in BMMs. As expected, the majority of miR-149-5p was sequestered in the cytoplasm, and the number of fluorescent foci increased after ZA treatment and decreased after JSH-23 treatment, (Figure 4I). Next, we set to examine the expression of the primary transcript of miR-149 (pri-miR149). In line with FISH assay results, a similar trend was observed in qRT-PCR (Figure 4H). These results indicated that p65 might regulate miR-149 expression at the transcription level. Next, we analyzed the putative binding sites of p65 in the pri-miR149 promoter and found one predicted binding region with a high-affinity score. Primers were designed accordingly, and a ChIP assay was performed using a specific p65 antibody. The qRT-PCR and results showed significant enrichment of p65 in this binding region compared with IgG control (Figure 4J). Furthermore, we constructed luciferase reporters based on the putative promoter region and found that the luciferase activity of WT vectors was increased by ZA treatment and decreased by JSH-23 treatment, whereas this effect could be eliminated using mutant vectors (Figure 4K). Taken together, these results indicate that ZA can activate p65 to promote miR-149-5p transcription and its subsequent loading in BMM-derived EVs.

### miR-149-5p–loaded EVs suppress biological functions of ECs via Rap1a/Rap1b/VEGFR2 signaling.

**Figure 5:**
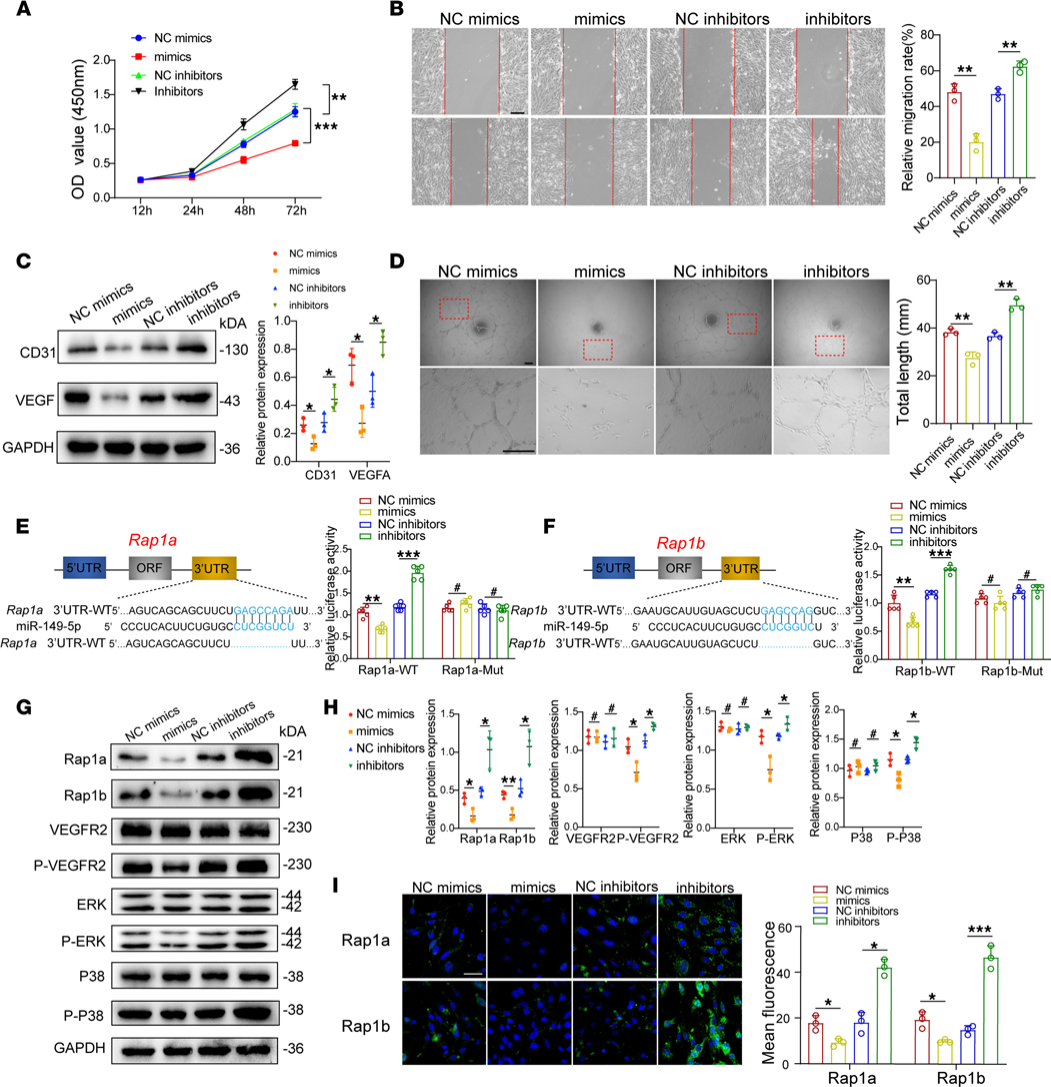
miR-149-5p directly targets *Rap1a/Rap1b* and regulates the VEGFR2 pathway. (**A**) CCK8 assay analysis showing EC proliferation after transfection with miR-149-5p mimics or inhibitors. *n* = 3. (**B**) Representative images of wound scratch assay of ECs and quantification analysis. Scale bars: 100 μm. *n* = 3. (**C**) Western blot and semiquantitative analysis of CD31 and VEGF expression of ECs. *n* = 3. (**D**) Representative images of tube formation assay of ECs and quantification analysis. Scale bars: 100 μm. *n* = 3. (**E**) Identification of miR-149-5p binding sites on the 3′-UTR of *Rap1a* by bioinformatics analysis and dual-luciferase reporter assays. *n* = 5. (**F**) Identification of miR-149-5p binding sites on the 3′-UTR of *Rap1b* by bioinformatics analysis and dual-luciferase reporter assays. *n* = 5. (**G**) Western blot analysis of proteins related to the Rap1a/Rap1b/VEGFR2 pathway. (**H**) Semiquantitative analysis of Western blot in **G**. *n* = 3. (**I**) Representative immunostained images of the expression of Rap1a and Rap1b in ECs after transfection with miR-149-5p mimics or inhibitors and quantitative analysis. Scale bars: 100 μm. *n* = 3. Results are presented as the mean ± SD. *P < 0.05; **P < 0.01; ***P < 0.001; ^#^*P* > 0.05 by Student’s *t* test.

To further investigate the role of miR-149-5p–loaded EVs in ECs function, ECs were treated with miR-149-5p mimics or inhibitors, and results showed that miR-149-5p mimics significantly inhibited EC proliferation, while miR-149-5p inhibitors improved EC proliferation (Figure 5A). Moreover, miR-149-5p mimics suppressed ECs migration, whereas miR-149-5p inhibitors promoted ECs migration (Figure 5B and Supplemental Figure 2A). Consistent with the above phenotypes, the expression of angiogenesis-related proteins CD31 and VEGFA decreased in miR-149-5p mimics group and increased in miR-149-5p inhibitors group (Figure 5C). Notably, miR-149-5p mimics inhibited tube formation of ECs, while miR-149-5p inhibitors exerted the opposite effect (Figure 5D). Collectively, our data suggest that miR-149-5p suppresses the biological functions of ECs.

We next conducted bioinformatics analysis to search for miR-149-5p target candidates involved in cellular functions of ECs. The predicted results showed that 2 highly homologous small G proteins, Rap1a and Rap1b, were the direct targets of miR-149-5p and were required for physiological functions of ECs via the VEGFR2 signaling pathway (36–38). To determine the *Rap1a* and *Rap1b* mRNA binding sites for miR-149-5p, we constructed WT and mutant vectors with 3′-UTRs of the respective genes for dual-luciferase reporter assay. Cellular luciferase activity markedly increased in miR-149-5p inhibitor group, whereas it decreased in miR-149-5p mimics group (Figure 5, E and F). Furthermore, the protein levels of Rap1a and Rap1b as well as VEGFR2 phosphorylation, ERK phosphorylation, and P38 phosphorylation were all decreased in mimics group but elevated in inhibitors group (Figure 5, G and H), and immunostaining results also showed a similar tendency (Figure 5I). Thus, miR-149-5p suppresses biological functions of ECs via the Rap1a/Rap1b/VEGFR2 signaling axis.

### miR-149-5p directly binds to the 3′-UTR of Traf6 and impedes osteoclast differentiation.

**Figure 6:**
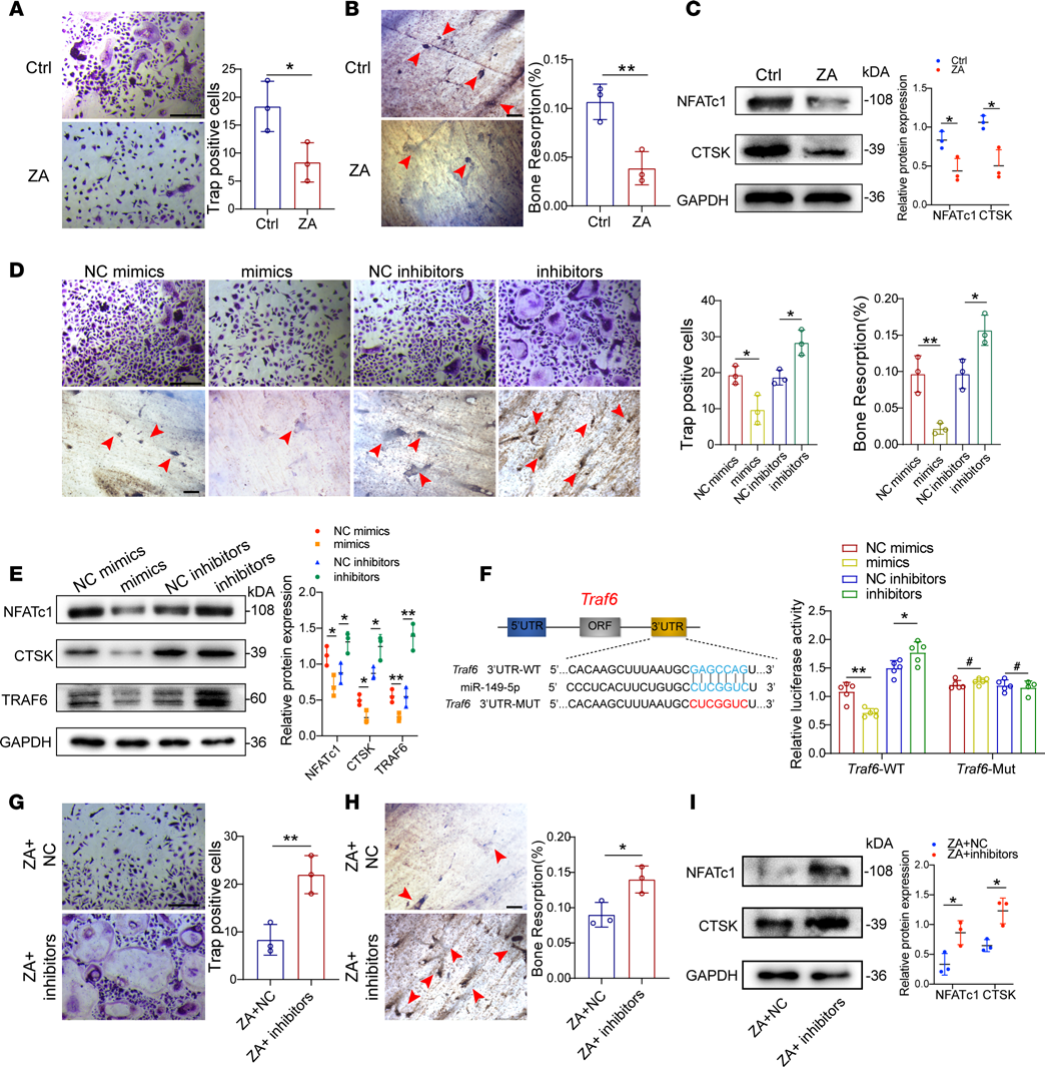
miR-149-5p directly binds to the 3′-UTR of *Traf6* and impedes osteoclast differentiation. (**A**) TRAP staining showing osteoclast formation after treatment with PBS or ZA. Scale bars: 100 μm. *n* = 3. (**B**) Wheat germ agglutinin staining showing bone resorption pits (red arrowheads) after treatment with PBS or ZA. Scale bars: 100 μm. *n* = 3. (**C**) Western blot and semiquantitative analysis of NFATc1 and CTSK expression. *n* = 3. (**D**) TRAP staining and bone resorption pits (red arrowheads) assay of osteoclasts after transfection with miR-149-5p mimics or inhibitors. Scale bars: 100 μm. *n* = 3. (**E**) Western blot and semiquantitative analysis of NFATc1, CTSK, and TRAF6 expression. *n* = 3. (**F**) WT and mutant *Traf6* vectors were subjected to dual-luciferase reporter assays. *n* = 5. (**G**) TRAP staining showing osteoclast formation after treatment with ZA+NC inhibitors (ZA and miR-149-5p negative control) or ZA+ inhibitors (ZA and miR-149-5p inhibitors). Scale bars: 100 μm. *n* = 3. (**H**) Wheat germ agglutinin staining showing bone resorption pits (red arrowheads) after treatment with ZA+NC inhibitors or ZA+ inhibitors. Scale bars: 100 μm. *n* = 3. (**I**) Western blot and semiquantitative analysis of NFATc1 and CTSK expression. *n* = 3. Results are presented as the mean ± SD. *P < 0.05; **P < 0.01; ^#^*P* > 0.05 by Student’s *t* test.

To elucidate the potential regulatory roles of miR-149-5p in osteoclast formation and differentiation, we investigated the osteoclastic differentiation of BMMs and found that ZA remarkably inhibited the formation of multinucleated osteoclasts and their bone resorption effect (Figure 6, A and B). Meanwhile, the expression of osteoclast-related proteins NFATc1 and CTSK decreased in ZA-treated group (Figure 6C). Then, we further verified that miR-149-5p mimics also inhibited the formation of osteoclasts, reduced resorption pit area, and decreased the expression of osteoclast-related proteins. In comparison, the opposite trends were observed in the inhibitor-treated group (Figure 6, D and E). Bioinformatics analysis predicted *Traf6*, which has been implicated in several molecular pathways related to osteoclastogenesis (39, 40), as another putative target gene of miR-149-5p. The expression of TRAF6 was significantly reduced or increased after transfection with miR-149-5p mimics or inhibitors, respectively (Figure 6E). To further ascertain whether miR-149-5p could directly bind to the *Traf6* 3′-UTR, we constructed WT and mutant vectors of *Traf6* and employed a dual-luciferase reporter assay. Indeed, miR-149-5p mimics and inhibitors remarkably affected luciferase activities after cotransfection with WT vector, while the effect was absolutely eliminated when the mutant vector was cotransfected (Figure 6F). Furthermore, we also investigated whether miR-149-5p could influence macrophage polarization. The results revealed that miR-149-5p mimics or inhibitors could change the polarization of BMMs to M1 or M2 polarization, respectively (Supplemental Figure 2, B and D). More importantly, the miR-149-5p inhibitor could reverse the inhibition effect of ZA on multinucleated osteoclast formation, bone resorption, and the expression of osteoclast-related proteins (Figure 6, G–I). Therefore, these findings suggest that miR-149-5p directly binds to the *Traf6* 3′-UTR and impedes osteoclastic differentiation and function.

### Local delivery of antagomiR-149-5p prevents BRONJ in mice.

**Figure 7:**
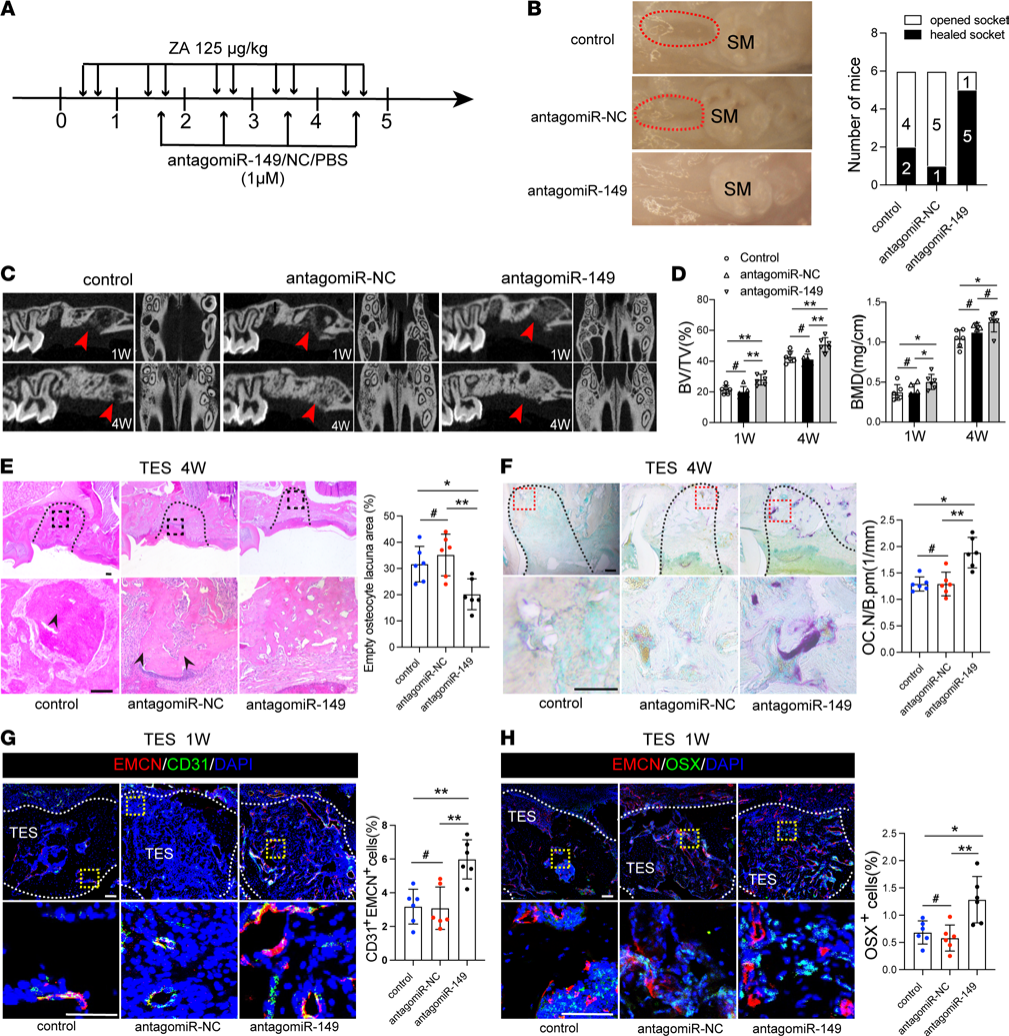
Local delivery of antagomiR-149-5p prevents BRONJ in mice. (**A**) Time schedule of the local delivery of PBS (empty vector control), antagomiR-NC, and antagomiR-149-5p into BRONJ model mice. Numbers indicate treatment days. (**B**) Clinical appearance of the gingival mucosa at TES. Red dotted lines outline the open socket with exposed bone adjacent to the second molar. The number of mice with the open socket was quantified. (**C**) Micro-CT analysis of new bone fill in TES. (**D**) Quantitative measurements of BV/TV and BMD for new bone in TES. *n* = 6. (**E**) Representative H&E-stained images of TES 4 weeks after tooth extraction. Scale bars: 100 μm. *n* = 6. (**F**) Representative TRAP staining showing osteoclastic activity and quantification of osteoclast numbers in TES. Scale bars: 100 μm. *n* = 6. (**G**) Representative immunostained images of EMCN^hi^CD31^hi^ vessels in TES from antagomiR-NC and antagomiR-149-5p mice. Scale bars: 100 μm. *n* = 6. (**H**) Representative immunostained images of osterix^+^ perivascular osteoprogenitors. Scale bars: 100 μm. *n* = 6. Results are presented as the mean ± SD. *P < 0.05; **P < 0.01; ^#^*P* > 0.05 by 1-way ANOVA.

Having identified the role of miR-149-5p on angiogenesis and bone remolding, we next wanted to determine the therapeutic value of miR-149-5p inhibition on BRONJ in vivo. We constructed antagomiR-149-5p and used a local injection method to deliver it into the local mucosa surrounding the TES of BRONJ mice to observe its therapeutic effects (Figure 7A). AntagomiR-149-5p mice showed nearly complete oral socket wound healing (5 of 6 mice), whereas 66.7% of control mice (4 of 6 mice) and 83.3% of their antagomiR-NC (negative control) counterparts (5 of 6 mice) exhibited open extraction sockets (Figure 7B). Micro-CT analysis revealed better TES healing in antagomiR-149-5p mice compared with control and antagomiR-NC counterparts (Figure 7, C and D). Similar findings could be found with histopathological analysis, and the results showed that antagomiR-149-5p mice had a lower percentage of socket necrotic bone area than their control and antagomiR-NC counterparts (Figure 7E). Additionally, TRAP staining revealed that local administration of antagomiR-149-5p could contribute to a remarkable increase of osteoclasts in TES (Figure 7F), indicating an active bone remodeling in jaw bone healing.

Furthermore, to investigate whether local miR-149-5p inhibition could contribute to skeletal microvasculature, we also explored the change of type H vessels and found a higher number of CD31^hi^EMCN^hi^ cells in antagomiR-149-5p mice compared with that in their control and antagomiR-NC counterparts (Figure 7G). More OSX^+^ cells surrounding EMCN^+^ vessels could be observed in antagomiR-149-5p mice compared with that in the controls (Figure 7H). Taken together, these findings suggest that local delivery of antagomiR-149-5p may prevent BRONJ.

## Discussion

**Figure 8:**
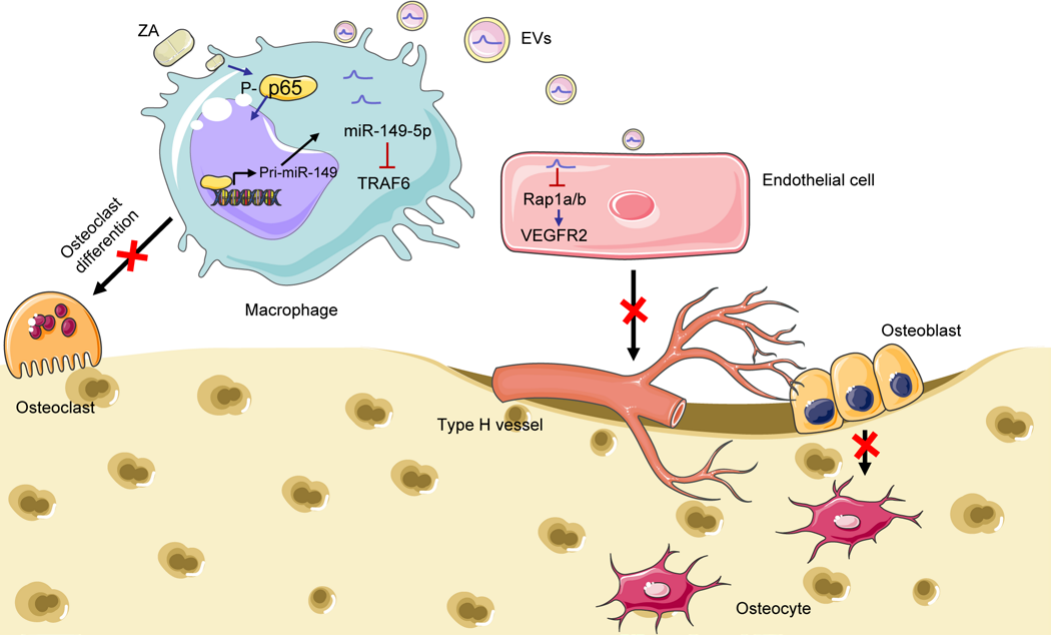
Working model of regulation of skeletal angiogenesis and bone remolding in BRONJ by macrophage miR-149-5p. ZA activated the NF-κB signaling pathway and induced p65 nuclear translocation in bone marrow–derived macrophages (BMMs) to promote miR-149-5p transcription, resulting in inhibited osteoclast differentiation via direct binding to the *Traf6* 3′-UTR region. Moreover, miR-149-5p–loaded EVs derived from ZA-treated BMMs could regulate skeletal angiogenesis via the Rap1a/Rap1b/VEGFR2 pathway in BRONJ.

In this study, we established a BRONJ mouse model to investigate the pathophysiology of BRONJ and found that ZA could impair skeletal angiogenesis in TES in vivo. At the molecular level, ZA activated NF-κB signaling and induced p65 nuclear translocation in macrophages, leading to a promotion of miR-149-5p transcription. Then, overexpressed miR-149-5p directly targeted *Traf6* 3′-UTR, resulting in the inhibition of osteoclast differentiation. At the cellular level, miR-149-5p–loaded BMM-derived EVs were found to shuttle to ECs and subsequently suppressed the biological functions of ECs via the Rap1a/Rap1b/VEGFR2 pathway (Figure 8). For diagnostic application, miR-149-5p was abundant in serum samples of both BRONJ mice and patients with BRONJ. Therefore, the level of mature miR-149-5p transcripts in serum samples may serve as a potential diagnostic marker for BRONJ. In the therapeutic perspective, inhibition of miR-149-5p expression significantly inhibited the occurrence of BRONJ in the rodent model, suggesting miR-149-5p as a potential therapeutic target in the treatment of BRONJ.

Angiogenesis, especially of type H vessels, is intimately coupled with osteogenesis during skeletal development and bone regeneration (41–43). Blood vessels not only provide bone tissues with the necessary nutrients, oxygen, and growth factors, but also play a vital role in bone regeneration during the bone healing processes (44, 45). Recently, Yan et al. found that type H vessels also exist in alveolar bone, with a unique morphology and spatial distribution pattern (22). These special subtypes of vessels arise from the cancellous alveolus plate, lining with Sharpey’s fibers. In this study, we found that the abundance of CD31^hi^EMCN^hi^ type H vessels was significantly decreased in the TES of BRONJ mice. Additionally, reduction of OSX^+^ perivascular osteoprogenitors could also be observed in BRONJ mice (Figure 1, G and H). It has been reported that the abundance of osteogenic CD31^+^ vasculature in femurs is unaffected by treatment with ZA, which could be explained by different concentration and distribution kinetics of ZA in jaw bone and appendicular bones (46). The mouse EC line C166 was used in our in vitro experiments to recapitulate the in vivo findings. We found that ZA was mostly endocytosed by BMMs rather than ECs. More importantly, biological functions of ECs were seemed not affected by ZA treatment but inhibited when cocultured with ZA-treated BMMs, suggesting an indirect effect of ZA on ECs via BMM/ECs intercellular communications.

Many studies have supported the roles of EV-encapsulated miRNAs in intercellular communication through paracrine signaling in macrophage-related diseases. As the main essential component of EVs, miRNA has attracted much attention in EV functional studies. Wang et al. (47) have reported that exosomes derived from M1 macrophages aggravate neointimal hyperplasia following carotid artery injuries in mice through the miR-222/CDKN1B/CDKN1C pathway. Xiong et al. (48) have found that M2 macrophage–derived exosomal miRNA‑5106 induces bone mesenchymal stem cells toward osteoblastic fate by targeting salt‑inducible kinase 2 and 3. Additionally, M1‑like macrophage‑derived EVs have been found to suppress angiogenesis and exacerbate cardiac dysfunction in a myocardial infarction microenvironment (49). In this study, ZA-treated BMM-derived EVs were found to transfer regulatory components into ECs that inhibit proliferation, migration, and tube formation, which were attributed to the high expression level of miR-149-5p. Moreover, we demonstrated that miR-149-5p overexpression was the key player in the modulation of osteoclast differentiation from BMMs by directly inhibiting *Traf6* expression, which has been linked to the RANK/RANKL pathway (39, 40). Therefore, these findings are likely to extend the current understanding of the inhibitory roles of ZA on osteoclastic functions. Notably, macrophage polarization could also be altered by modulating the miR-149-5p expression in our study (Supplemental Figure 2). However, we have not identified the miR-149-5p target related to macrophage polarization. Thus, the underlying regulatory mechanism linking miR-149-5p and macrophage polarization needs to be further investigated.

We further explored the preventative and therapeutic value of miR-149-5p by locally administering chemically modified antagomiR-149-5p into the local mucosa surrounding the TES of BRONJ mice, which subsequently accelerated the healing of TES, promoted skeletal angiogenesis, and increased the number of OSX^+^ perivascular osteoprogenitors. Several studies have revealed that genetic factors or miRNA levels could be used to establish the diagnostic model for BRONJ (50–53). Intriguingly, our findings also reinforced the notion that circulating miR-149-5p might be a diagnostic marker for BRONJ. However, large clinical samples are required to verify whether it could serve as an early detection method to assess the risk of BRONJ.

Collectively, our study highlights the critical role of macrophage-derived EVs in regulating EC biological functions and skeletal angiogenesis in response to ZA administration. Our results suggest that miR-149-5p–loaded BMM-derived EVs can shuttle into ECs where they abrogate the biological functions of ECs. Beyond the intercellular role, ZA-induced increased miR-149-5p transcription inhibited osteoclastic differentiation, suggesting that targeting miR-149-5p might be a therapeutic strategy for TES bone regeneration in BRONJ.

## Methods

### Mouse model.

Eight-week-old male C57BL/6J mice were purchased from Nanjing Medical University. Mice were allocated into cages randomly, with 4–5 mice per standard cage housed at 22°C–25°C with unlimited water and rodent chow. The BRONJ-like models in mice were established as described previously (26, 54). Briefly, mice were intravenously injected with ZA (125 μg/kg, Novartis Oncology) or vehicle solution (PBS) biweekly via tail veins for 5 consecutive weeks (*n* = 6 per group). The dose of 125 μg/kg is almost 2-fold higher than the oncologic ZA dose of 66 μg/kg (55), and it is the average of BP doses used in other animal models (56). We used this dose to increase the incidence of BRONJ in our animals, because there appears to be a dose-dependent effect of BPs on BRONJ incidence in humans (57). A total of 10 doses of ZA were intravenously administered for 5 consecutive weeks. The first right maxillary molar teeth were extracted 1 week after the initial injection of ZA, which was performed under deep anesthesia via the intraperitoneal injection of ketamine (100 mg/kg). To explore the effect of miR-149-5p inhibition in preventing BRONJ, C57BL/6J mice were divided into 3 groups randomly; mice in each group were respectively injected with PBS (empty vector control), antagomiR-NC (1 μM), or antagomiR-149-5p (1 μM) at a dose of 10 μL into the mesial and distal mucosa of the buccal and palatal sides of the maxillary TES using a microsyringe once a week after tooth extraction under deep anesthesia (58). miRNA antagomiR-149-5p and antagomir-NC were synthesized by GenePharma Co. Ltd. and were specially chemically modified to directly penetrate the cell, which possess high stability and can maintain activity for about 4 weeks in TES. The maxillae were harvested after 1 or 4 weeks after the extraction of teeth for further analysis (*n* = 6 per group).

### Micro-CT.

The microarchitectural properties of the maxillae were analyzed using a micro-CT scanning system (Skyscan 1176). The maxillae were scanned at a high resolution (18 μm), with an energy of 55 KV and 456 μA. Three-dimensional reconstructions of the maxillae were acquired and analyzed using NRecon v1.6 and CTAn v1.13.8.1 software. The region of interest was defined to focus on the TES of the first maxillary molar teeth. The healing of the TES was analyzed by the 2 following parameters: BV/TV and BMD.

### Histologic analysis and immunofluorescence staining.

The mouse maxillae tissues were fixed in ice-cold 4% PFA and decalcified with 10% EDTA at 4°C for 14 days. All samples were embedded in OCT compound (Sakura) and were prepared in 4-μm-thick sections for H&E and immunofluorescence staining. According to our previous studies, for necrotic bone area analysis, the empty osteocyte lacuna area, as a percentage of total view area, was counted in at least 3 slices of the TES per mouse. Immunofluorescence staining was performed as described previously (26). After treatment with 1% Triton X-100 for 15 minutes, sections were blocked with goat serum at room temperature for 30 minutes and incubated with primary antibodies at 4°C overnight. Then, maxillae sections were incubated with Cy3- or FITC-labeled secondary IgG for 1 hour. Finally, they were stained with DAPI for 1 minute and visualized under a confocal fluorescence microscope. Semiquantitative analysis was performed on at least 3 sections of each sample. Positive signals in at least 3 random fields per section were analyzed and expressed as the percentage of total nucleic cells. Information on antibodies used in this study is listed in Supplemental Table 2.

### Cell culture and differentiation.

The culture and differentiation of BMMs were described in our previous study (26). Briefly, bone marrow cells were flushed out of bone marrow cavities of femurs and tibias of C57BL/6 mice and cultured with DMEM containing 2% FBS and antibiotics (100 U/ml penicillin and 100 mg/ml streptomycin) for 4 hours. Then, all nonadherent cells were collected and reseeded in complete DMEM containing 10% FBS, antibiotics, and murine M-CSF (10 ng/ml) for 3 days and changed with fresh media every 3 days. For the differentiation of osteoclasts, the BMMs were cultured with complete DMEM supplemented with RANKL (20 ng/ml). C166 cells, a mouse EC line, were purchased from the iCell Bioscience Inc. and cultured in DMEM containing 10% FBS and antibiotics.

### Immunofluorescence.

To determine whether ZA was internalized by BMMs or ECs, the BMMs and ECs were seeded on coverslips and treated with 10 μM of the 5-FAM-ZA (BV111001, BioVinc). Then, the cells were fixed with 4% PFA and examined under a confocal fluorescence microscope (Zeiss LSM 710). EVs derived from BMMs were labeled with DiI (Thermo Fisher Scientific) according to the manufacturer’s instructions. Briefly, 1 μM DiI was added into EVs, which were dissolved in PBS and incubated at 37°C for 20 minutes. Subsequently, DiI-labeled EVs were incubated with ECs. Finally, after being fixed with 4% PFA for 15 minutes, ECs were stained with DAPI for 1 minute and examined under a confocal fluorescence microscope. For detection of the expression of Rap1a and Rap1b, ECs were seeded on coverslips for 24 hours at 37°C, fixed with 4% PFA for 15 minutes, and permeabilized with 1% Triton X-100. ECs were incubated with primary antibodies overnight at 4°C after preincubation with goat serum. Then, ECs were incubated with Cy3- or FITC-labeled secondary IgG for 1 hour. Finally, coverslips were stained with DAPI for 1 minute and visualized under a confocal fluorescence microscope.

### CCK8 assay.

The proliferation of ECs with different treatments was assessed using Cell Counting Kit-8 (CCK8; K1018, APExBIO)). In brief, ECs were seeded at an initial density of 5 × 10^3^ cells/well in 96-well plates. After different treatments, 10 μL CCK8 solution was added into each well of the palate for 12, 24, 48, and 72 hours. Then, the palate was incubated for 3 hours. The optical density value was assessed by a microplate scanning reader (BioTek Instruments) at 450 nm.

### Cell migration assay.

Wound scratch assay and Transwell migration assay were performed according to our previous studies (59). In brief, ECs were seeded in a 6-well plate at the 80% confluence. After different treatments, a sterile 200 μL pipette tip was used to form an artificial wound on the cell monolayer. The suspended ECs were washed thoroughly with PBS. The wounds were photographed at 0 or 12 hours, and the results were quantified using ImageJ software (NIH).

For the Transwell migration assay, 5 × 10^4^ ECs were suspended in 200 μL serum-free DMEM and seeded in Transwell inserts. Complete DMEM medium was added in lower wells to stimulate migration. After incubation at 37°C for 12 hours, the migrated cells in the bottom side were stained with Crystal Violet dye.

### Tube formation assay.

To evaluate the ability of ECs, tube formation assays were performed according to our previous studies (60). In brief, ECs were seeded in the ECMatrix gel (BD Biosciences) at the density of 10^3^ cells/well, and vessel-like structures were observed after 12 hours. Tube length was quantified using ImageJ software.

### Western blot.

Western blot was performed as described in our previous studies (26). In brief, total protein lysates were obtained from harvested cells in RIPA lysis buffer (Beyotime). Total proteins were separated on 10% SDS-PAGE gels and transferred to 0.45 μm PVDF membranes (Millipore). Membranes were blocked in 5% BSA for 2 hours and incubated with primary antibodies at 4°C overnight. After washing with Tris-buffered saline with Tween-20 3 times, membranes were incubated with secondary antibodies for 1 hour at room temperature and visualized by chemiluminescence. Semiquantitative measurements were performed using ImageJ software. The expression protein levels were semiquantified as the ratio of the level of the protein of interest to the level of GAPDH in each group. Information regarding antibodies used in this study is listed in Supplemental Table 2.

### Quantitative real-time PCR analysis.

RNA was extracted from BMMs using a RNA isolation kit (Takara) according to the manufacturer’s instructions. A quantitative RT-PCR analysis was performed using the SYBR Green PCR Master Mix (Takara Bio). miR-149-5p in EVs from BMMs supernatant or serum of mice was extracted using the miRNeasy Serum/Plasma kit (QIAGEN). The expression of miR-149-5p was detected with the miRNA qRT-PCR Detection Kit (GeneCopoeia). Levels of each mRNA or miRNA, respectively, were normalized to the GAPDH or U6 levels. The miR-149-5p level of EVs was compared with spiked-in ce-miR-39 to normalize miRNA expression, which was used as the reference by using the miRNeasy Serum/Plasma Spike-In Control kit (QIAGEN). Each experiment was performed at least 3 times. The 2^−ΔΔCT^ method was used to quantify expression of the genes of interest. The primer sequences used in this study are listed in Supplemental Table 1.

### Isolation and identification of EVs.

EVs derived from BMMs were collected according to our previous study (61). The supernatant of BMMs was collected and centrifuged at 300*g* for 15 minutes, 2,000*g* for 20 minutes, and 10,000*g* for 30 minutes to remove cell debris and large EVs. Then, the reminding supernatant of BMMs was centrifuged 100,000*g* for 70 minutes twice to remove the possible contaminating protein. The pellets were successfully resuspended in PBS and stored at −80°C. A transmission electron microscope (Hitachi) was used to view EVs morphologies. The nanoparticle tracking analysis was performed to determine the size distribution of BMM-derived EVs by ZetaView Nanoparticle Tracking Analyzer (PMX).

### Transfection of miR-149-5p mimics and inhibitors.

miR-149-5p mimics, inhibitors, and negative control (50 nM) were transfected into cells using Lipofectamine 2000 (Invitrogen) and synthesized by GenePharma Co. Ltd. Cells were harvested and analyzed 48 hours after transfection.

### miRNA-Seq.

For miRNA-Seq of BMM-derived EVs, total RNA samples were reversed into indexed cDNA sequencing libraries with the Small RNA Library Prep Kit for Illumina (NEB). First, the 3′ hydroxy of miRNAs was added with a single-stranded adenylated DNA adapter using Small RNA 3 ADT (NEB). A 5′ adapter was added to the 5′ phosphate using Small RNA 5 ADT. After adapter ligation, single-stranded cDNA was obtained through a reverse-transcription reaction. Next, cDNA was mixed with Small RNA index Primer (NEB) using the HANTAI SmallRNA Library Index Kit, and PCR amplification was performed. PAGE gel was used for electrophoresis fragment screening purposes to get small RNA libraries. Finally, PCR products were purified (AMPure XP System), and library quality was assessed on the Agilent Bioanalyzer 2100 system. The clustering of the index-coded samples was performed on a cBot Cluster Generation System. The library preparations were sequenced on an Illumina platform, and reads were generated after cluster generation. The miRNA-Seq data have been deposited in the NCBI database (PRJNA843915).

### TRAP staining and bone resorption pit assay.

Mature osteoclasts were fixed in 4% PFA for 15 minutes and stained with the TRAP kit (MilliporeSigma, 387A-1KT) according to the manufacturer’s instructions. Three or more positively stained nuclei were identified as mature osteoclasts. For the bone resorption pit assay, BMMs were cultured on bovine bone slides for differentiation into osteoclasts. After 7 days, bovine bone slices were washed with PBS and incubated with wheat germ agglutinin (20 μg/mL, MilliporeSigma). Bone slides were then stained with diaminobenzidine and imaged with the Leica Application Suite.

### Dual-luciferase reporter assay.

293T cells or BMMs were seeded in 24-well plates at an initial concentration of 2 × 10^5^ cells/well. After culture for 24 hours, they were cotransfected with pGL3-basic reporter vector, Renilla vector, and miR-149-5p mimics or inhibitors using Lipofectamine 2000. Luciferase activities were measured by the Dual-Luciferase Reporter Assay Kit (Vazyme) after 48 hours of transfection.

### ChIP assay.

Chip assay was carried out using the SimpleChIP Plus Sonication ChIP kit (Cell Signaling Technology). In brief, cells were first fixed with formaldehyde, which was a reversible protein-DNA cross-linking agent. Then, cells were lysed, and chromatin was harvested and fragmented using sonication. The chromatin was then subjected to immunoprecipitation using p65 antibody. After immunoprecipitation, the protein-DNA cross-links were reversed, and the DNA was purified. The enrichment of putative binding sites by p65 in pri-miR-149 promoter was detected by qRT-PCR.

### FISH.

miR-149-5p probe was designed and synthesized by Genepharma. The subcellular localization of miR-149-5p was examined with the FISH kit (Genepharma) according to the manufacturer’s instructions. In brief, BMMs were fixed with 4% PFA for 20 minutes and permeabilized with 1% Triton X-100. Then, the coverslips were incubated with miR-149-5p FISH probe (5′-GGGAGTGAAGACACGGAGCCAGA-3′) diluted in hybridization buffer overnight at 37°C. After staining with DAPI, the coverslips were visualized under a confocal fluorescence microscope.

### Flow cytometry assay.

Flow cytometry assay was performed as described in one of our previous studies (26). Briefly, single-cell suspensions from BMMs were prepared; cells were fixed and permeabilized using a BD Fixation/Permeabilization Kit (554715) and treated with Fc block reagent (BD Pharmingen, 553141). Then, the cells were stained with CD11b, CD86, and CD206. Information about antibodies used in flow cytometry is listed in Supplemental Table 2. A FACSVerse flow cytometer (BD Biosciences) was used, and the data analysis was performed using FlowJo v.10 software (BD Biosciences).

### Statistics.

All results are presented as the mean ± SD from at least 3 independent experiments. During the analysis period, each sample’s identity information was sealed to avoid bias, and each value was checked twice by 2 examiners. Statistical analysis was performed using GraphPad Prism 8 software. Statistical significance for 2-group comparisons was assessed using 2-tailed unpaired Student’s *t* test. Statistical significance of differences among more than 2 groups was calculated using 1-way ANOVA with Tukey’s post hoc test. P values of less than 0.05 were considered significant.

### Study approval.

All animal experimental procedures were conducted according to guidelines approved by the Animal Care Committee of Nanjing Medical University (no. 1805006).

## Author contributions

XS performed experiments, analyzed data, and wrote the manuscript. WZ contributed to the design, data acquisition, and interpretation and critically revised the manuscript. PZ contributed to the conception, design, and data acquisition. YF helped perform experiments. JC and LL contributed to the critical revision of the manuscript. RX contributed to the design and data acquisition and revised the manuscript. HJ conceived of this study, analyzed the data, and revised the manuscript.

## Supplementary Material

Supplemental data

## Version 1 08/22/2022

Electronic publication
