# Supplementary material for: Macrophage miR-149-5p induction is a key driver and therapeutic target for BRONJ
Source: JCI Insight. 2022 Aug 22;7(16):e159865. doi: 10.1172/jci.insight.159865 (PMC9462481; doi:10.1172/jci.insight.159865)
Supplement: Supplemental data [file jciinsight-7-159865-s118.pdf]

Supplemental Information

Table S1. Primer sequence for qRT-PCR, ChIP qPCR.

Gene	Primer Sequence (5' - 3')
GAPDH	F: AGGTCGGTGTGAACGGATTTG<br>R: TGTAGACCATGTAGTTGAGGTCA
U6	ATGGACTATCATATGCTTACCGTA
miR-149-5p	CTCCGTGTCTTCACTCCCAAA
miR-16-1-3p	AGTATTGACTGTGCTGCTGAAAA
miR-23a-3p	GCCAGGGATTTCCAAAA
miR-145-3p	AGTTTTCCCAGGAATCCCTAAA
miR-129-5p	TGCGGTCTGGGCTTGCAAA
miR-221-3p	TACATTGTCTGCTGGGTTTCAAA
Pri-miR149	F: AATGCATGGGCTTCTGGGC<br>R: CTCCCTCCTCGGACAAACAC
Primer for Pri-miR149 Promotor (ChIP-p65)	F: AGAGATTATACTCCGAAT<br>R: CACCTTCTGAAGATAATG
AntagomiR-149-5p	GGGAGUGAAGACACGGAGCCAGA
AntagomiR-NC	CAGUACUUUUGUGUAGUACAA

Table S2. Information of Antibodies

Antibodies (Dilution)	Source	Cat#
Rat monoclonal anti-Endomucin (1:100)	Santa Cruz	Cat# sc-65495
Goat polyclonal Goat anti- CD31 conjugated to Alexa Fluor 488 (1:100)	R&D Systems	Cat# FAB3628G
Rat polyclonal anti-Osterix (1:500)	Abcam	Cat# ab22552
Mouse monoclonal anti-CD31 (1:2000)	Proteintech	Cat# 66065-2-Ig
Rabbit monoclonal anti-CD31 (1:2000)	Proteintech	Cat# 19003-1-AP
Mouse monoclonal anti-CD63 (1:1000)	Abcam	Cat# ab213090
Rabbit anti-HSP70 (1:1000)	Cell Signaling Technology	Cat# 4872
Rabbit polyclonal anti-Calnexin (1:2000)	Proteintech	Cat# 10427-2-AP
Mouse monoclonal anti-GAPDH (1:1000)	Cell Signaling Technology	Cat# 5174
Rabbit monoclonal anti-p65 (1:1000)	Cell Signaling Technology	Cat# 8242

Mouse monoclonal anti-IκBα (1:1000)	Cell Signaling Technology	Cat# 4814
Rabbit monoclonal anti-Lamin B1 (1:1000)	Cell Signaling Technology	Cat# 13435
Rabbit polyclonal anti-Rap1a (1:2000)	Affinity	Cat# DF6157
Rabbit polyclonal anti-Rap1b (1:2000)	Proteintech	Cat# 10840-1-AP
Rabbit polyclonal anti-VEGFR2 (1:1000)	Proteintech	Cat# 26415-1-AP
Rabbit polyclonal anti-P-VEGFR2 (1:2000)	Affinity	Cat# AF4426
Rabbit monoclonal anti-ERK (1:1000)	Cell Signaling Technology	Cat# 4695
Rabbit monoclonal anti-P-ERK (1:1000)	Cell Signaling Technology	Cat# 4370
Rabbit anti-P38 (1:1000)	Cell Signaling Technology	Cat# 9212
Rabbit anti-P-P38 (1:1000)	Cell Signaling Technology	Cat# 9211
Mouse monoclonal anti-IκBα (1:1000)	Santa Cruz	Cat# sc-7294
Mouse monoclonal anti-CTSK (1:100)	Santa Cruz	Cat# sc-48353

Mouse monoclonal anti-TRAF6 (1:5000)	Proteintech	Cat# 66498-1-Ig
PE anti-mouse/human CD11b Antibody	BioLegend	Cat# 101208
APC Anti-Mouse CD206 Antibody	Elabscience	Cat# E-AB-F1135UE
FITC Anti-Mouse CD86	MULTI SCIENCE	Cat# AM08601

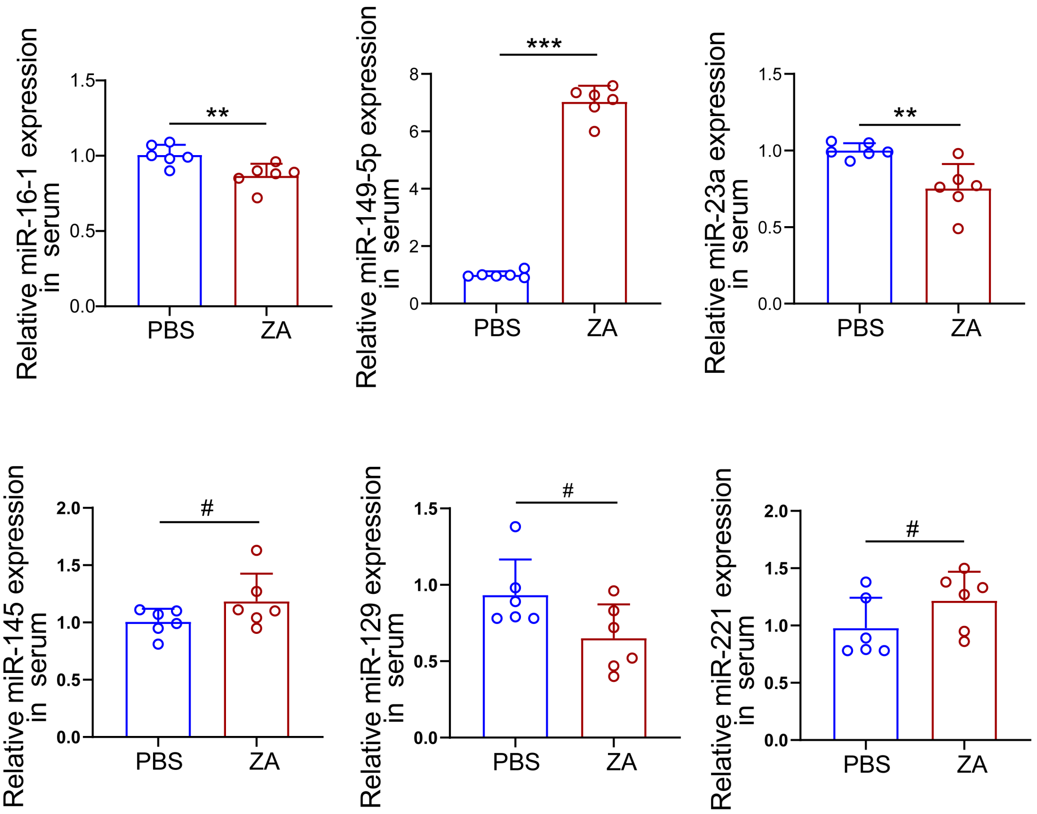

Fig S1: MiRNAs expression profiling in mice serum. Results are presented as the mean ± S.D. **$p < 0.01$; ***$p < 0.001$; #$p > 0.05$ by Student's t test.

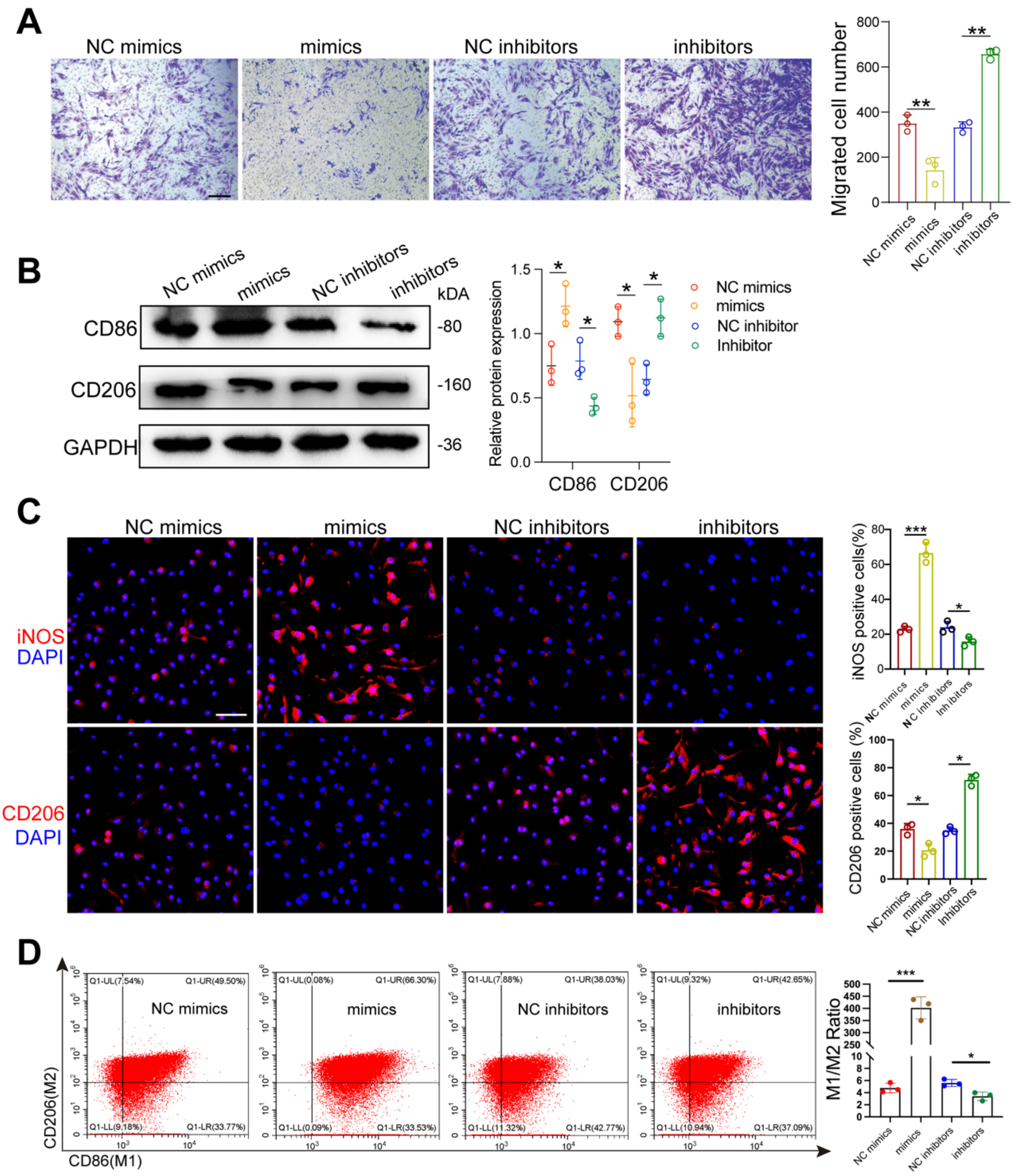

Fig S2: Effects of miR-149-5p on ECs migration and BMMs polarization. (A) Representative images of transwell migration assay of ECs and quantification analysis. Scale bars: 100 μm. n=3. (B) Western blot analysis of the expression of CD86 and CD206 in BMMs. n=3. (C) Representative immunostaining images of the expression of iNOS and CD206 in BMMs and quantification analysis. Scale bars: 100 μm. n=3. (D) Representative percentages of M1 (CD86) or M2 (CD206) macrophages were detected in by flow cytometric analysis. n=3. Results are presented as the mean ± S.D. *p < 0.05; ***p < 0.001 by Student's t test.
